# Biallelic *GRM7* variants cause epilepsy, microcephaly, and cerebral atrophy

**DOI:** 10.1002/acn3.51003

**Published:** 2020-04-14

**Authors:** Dana Marafi, Tadahiro Mitani, Sedat Isikay, Jozef Hertecant, Mohammed Almannai, Kandamurugu Manickam, Rami Abou Jamra, Ayman W. El‐Hattab, Jaishen Rajah, Jawid M. Fatih, Haowei Du, Ender Karaca, Yavuz Bayram, Jaya Punetha, Jill A. Rosenfeld, Shalini N. Jhangiani, Eric Boerwinkle, Zeynep C. Akdemir, Serkan Erdin, Jill V. Hunter, Richard A. Gibbs, Davut Pehlivan, Jennifer E. Posey, James R. Lupski

**Affiliations:** ^1^ Department of Molecular and Human Genetics Baylor College of Medicine Houston Texas 77030; ^2^ Department of Pediatrics Faculty of Medicine Kuwait University P.O. Box 24923 13110 Safat Kuwait; ^3^ Department of Physiotherapy and Rehabilitation School of Health Sciences Hasan Kalyoncu University Gaziantep 27000 Turkey; ^4^ Pediatric Metabolic and Genetics Division Tawam Hospital Al Ain Abu Dhabi United Arab Emirates; ^5^ Section of Medical Genetics Children's Hospital King Fahad Medical City Riyadh 11525 Saudi Arabia; ^6^ Division of Genetic and Genomic Medicine Nationwide Children’s Hospital Columbus Ohio; ^7^ Institute of Human Genetics University Medical Center Leipzig 04103 Leipzig Germany; ^8^ Department of Clinical Sciences College of Medicine University of Sharjah Sharjah 27272 United Arab Emirates; ^9^ Sheikh Khalifa Medical City (SKMC) P.O. Box: 51900 Abu Dhabi United Arab Emirates; ^10^ Human Genome Sequencing Center Baylor College of Medicine Houston Texas 77030; ^11^ Human Genetics Center University of Texas Health Science Center at Houston Houston Texas; ^12^ Center for Genomic Medicine Massachusetts General Hospital Boston Massachusetts; ^13^ Texas Children's Hospital Houston Texas 77030; ^14^ Department of Radiology Baylor College of Medicine Houston Texas 77030; ^15^ Section of Pediatric Neurology and Developmental Neuroscience Department of Pediatrics Baylor College of Medicine Houston Texas 77030; ^16^ Department of Pediatrics Baylor College of Medicine Houston Texas 77030

## Abstract

**Objective:**

Defects in ion channels and neurotransmitter receptors are implicated in developmental and epileptic encephalopathy (DEE). Metabotropic glutamate receptor 7 (mGluR7), encoded by *GRM7,* is a presynaptic G‐protein‐coupled glutamate receptor critical for synaptic transmission. We previously proposed *GRM7* as a candidate disease gene in two families with neurodevelopmental disorders (NDDs). One additional family has been published since. Here, we describe three additional families with *GRM7* biallelic variants and deeply characterize the associated clinical neurological and electrophysiological phenotype and molecular data in 11 affected individuals from six unrelated families.

**Methods:**

Exome sequencing and family‐based rare variant analyses on a cohort of 220 consanguineous families with NDDs revealed three families with *GRM7* biallelic variants; three additional families were identified through literature search and collaboration with a clinical molecular laboratory.

**Results:**

We compared the observed clinical features and variants of 11 affected individuals from the six unrelated families. Identified novel deleterious variants included two homozygous missense variants (c.2671G>A:p.Glu891Lys and c.1973G>A:p.Arg685Gln) and one homozygous stop‐gain variant (c.1975C>T:p.Arg659Ter). Developmental delay, neonatal‐ or infantile‐onset epilepsy, and microcephaly were universal. Three individuals had hypothalamic–pituitary–axis dysfunction without pituitary structural abnormality. Neuroimaging showed cerebral atrophy and hypomyelination in a majority of cases. Two siblings demonstrated progressive loss of myelination by 2 years in both and an acquired microcephaly pattern in one. Five individuals died in early or late childhood.

**Conclusion:**

Detailed clinical characterization of 11 individuals from six unrelated families demonstrates that rare biallelic *GRM7* pathogenic variants can cause DEEs, microcephaly, hypomyelination, and cerebral atrophy.

## Introduction

Defects in ion channels and neurotransmitter receptors are implicated in developmental and epileptic encephalopathies (DEEs) by impairing central nervous system (CNS) neurotransmission.^1^, ^2^ One such important group of receptors is the glutamate receptors. Glutamate is an excitatory neurotransmitter that has been extensively studied in epilepsy and that acts through two main receptor groups: ionotropic glutamate receptors (iGluRs) and metabotropic glutamate receptors (mGluRs).^3^ The iGluRs include N‐methyl‐D‐aspartate receptor (NMDAR), alpha‐amino‐3‐hydroxy‐5‐methyl‐4‐isoxazolepropionic acid receptor (AMPAR), and kainate receptor (KAR). Rare monoallelic and biallelic single nucleotide variants (SNVs) and copy number variants (CNVs) in genes encoding postsynaptic iGluRs subunits such as *GRIN1, GRIN2A*, *GRIN2B*, and *GRIN2D* encoding the NR1, NR2A, NR2B, and NR2D subunits of NMDAR, respectively, *GRIA2‐4* encoding the GluR2‐4 subunits of AMPAR, and *GRIK2* encoding the GluR6 subunit of KAR have been shown to cause a wide range of neurodevelopmental disorders (NDDs) and DEEs.^4^, ^5^, ^6^, ^7^, ^8^, ^9^, ^10^, ^11^

The mGluRs consist of eight receptors, mGluRs1‐8, encoded by *GRM1‐8,* that are classified into three groups based on sequence homology, ligand selectivity, and signal transduction mechanism.^12^ Group I consists of mGluR1 and mGluR5, group II consists of mGluR2 and mGluR3, and group III consists of mGluR4, mGluR6, mGluR7, and mGluR8. Groups II and III function presynaptically, whereas group I functions postsynaptically.^12^ Recent studies have focused on the role of mGluRs in neurodevelopmental and neuropsychiatric disorders. A genome‐wide CNV study using single nucleotide polymorphism (SNP) array revealed an enrichment of CNVs in *GRM1, 5, 7,* and *8* in subjects with attention deficit hyperactivity disorders versus apparently healthy controls.^13^ Another pilot case–control study supported an association between *GRM7* SNPs and autism spectrum disorder.^14^ Biallelic rare variants in *GRM1* have been shown to cause cerebellar ataxia.^15^

mGluRs are also involved in the pathophysiology of epilepsy.^16^ Agonists of postsynaptic group I mGluRs have proconvulsive properties while their antagonists have anticonvulsant activity in seizure models, and the opposite has been shown in the presynaptic mGluRs groups II and III. These findings are attributed to the role of group I mGluRs in enhancing neuronal excitability and of groups II and III in inhibiting hyperexcitability.^16^

mGluR7, encoded by *GRM7,* is the most highly conserved mGluR and is exclusively expressed in the CNS. mGluR7 functions as a constitutional dimer and consists of a binding domain, cysteine‐rich domain, transmembrane domain, and intracellular C‐terminus.^12^ mGluR7 plays a critical role in synaptic transmission by inhibiting further release of excitatory neurotransmitter glutamate and inhibitory neurotransmitter GABA when they reach high levels at the synapses.^17^ mGluR7‐knockout mice develop spontaneous stimulus‐provoked seizures, suggesting that disruption of *GRM7* expression may cause epilepsy.^18^

We previously proposed *GRM7* as a candidate gene for autosomal recessive NDD in four subjects from two unrelated families with NDD and rare biallelic missense variants.^19^ One additional family including two siblings with NDD and a rare homozygous stop‐gain allele in *GRM7* has been described since.^20^ Here, we describe five additional individuals from three unrelated families with biallelic variants in *GRM7* and a similar neurological DEE phenotype along with the six individuals previously reported with limited clinical data and provide evidence to support *GRM7* biallelic variants as a cause for NDDs, DEE, and microcephaly.

## Methods

### Participants and ethical approval

This study was approved by the Institutional Review Board (IRB) at Baylor College of Medicine under the Baylor Hopkins Center for Mendelian Genomics Protocol (IRB number H‐29697). All families except for Family 3 (MR005) were enrolled under protocol H‐29697. Family 3 (MR005) was enrolled under an IRB‐approved protocol at the University of Bonn (IRB number 033/08). For all research subjects, informed patient consent was obtained for publication of photographs.

### Exome sequencing, linkage analysis, variant interpretation, and phenotyping methods

**Figure 1:**
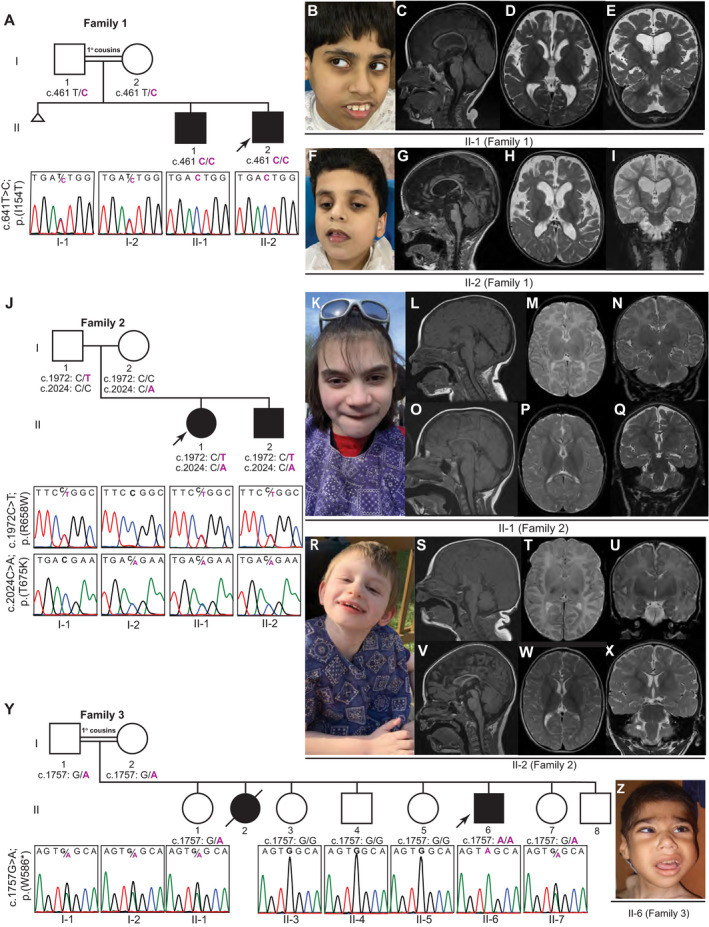
Pedigrees, Sanger sequencing, facial features, and brain MRI images of affected individuals from Families 1–3 with biallelic variants in *GRM7*. (A) Pedigree and Sanger sequencing showing segregation of the variants in *GRM7* in Family 1. (B) Facial photograph of individual II‐1 (Family 1) at 13 years showing prominent teeth and everted lower lip. (C‐E) Brain MRI of individual II‐1 (Family 1) at 3 years. T1‐weighted image (midsagittal view) shows moderate to severe thinning of corpus callosum (CC), excess cerebral spinal fluid (CSF) around the falx cerebri indicating severe cerebral volume loss, and minimal cerebellar volume loss (C). T2‐weighted images (axial and coronal views) show hypomyelination, particularly over the anterior temporal horns, with normal fourth ventricle and hippocampi (D and E). (F) Facial photograph of individual II‐2 (Family 1) at 10 years shows high forehead and hypotonic face (G‐I) Brain MRI of individual II‐2 (Family 1) at 2 years. T1‐weighted image (midsagittal view) showing foreshortening of CC, and excess CSF around the falx cerebri indicating severe cerebral volume loss (G). T2‐weighted images (axial and coronal views) show normally myelinated internal capsule, excessive subarachnoid spaces, enlarged lateral ventricles secondary to cerebral volume loss, and T2 hyperintensity in bilateral hippocampi (H and I). (J) Pedigree and Sanger sequencing showing segregation of the variants in *GRM7* in Family 2. (K) Facial photograph of individual II‐1 (Family 2) at 15 years shows a wide mouth. (L–N) Brain MRI of individual II‐1 (Family 2) at 2 months. T1‐weighted image (midsagittal view) shows normal CC (L). T2‐weighted images (axial and coronal views) show normal myelination for age (M and N). (O–Q) Brain MRI of individual II‐1 (Family 2) at 7 years. T1‐weighted image (midsagittal view) shows severe thinning of CC and normal cerebellum (O). T2‐weighted images (axial and coronal views) show global hypomyelination and normal hippocampus (P and Q). (R) Facial photograph of individual II‐2 (Family 2) at 10 years shows a wide mouth. (S–U) Brain MRI of individual II‐2 (Family 2) at day of life 2. T1‐weighted image (midsagittal view) shows normal CC (S). T2‐weighted images (axial and coronal views) show normal myelination for age (T‐U). (V‐X) Brain MRI of individual II‐2 (Family 2) at 5 years. T1‐weighted image (midsagittal view) shows severe thinning and foreshortening of CC, normal posterior pituitary and infundibulum, and normal vermis and cerebellum (V). T2‐weighted images (axial and coronal views) show global hypomyelination, small right cerebellar cyst and normal hippocampus (W and X). (Y) Pedigree and Sanger sequencing showing segregation of the variants in *GRM7* in Family 3. (Z) Facial features of individual II‐6 (Family 3) at 6 years showing thick lips, crowded teeth, low frontal hairline, remarkable nose, and bulbous nasal tip.

**Figure 2:**
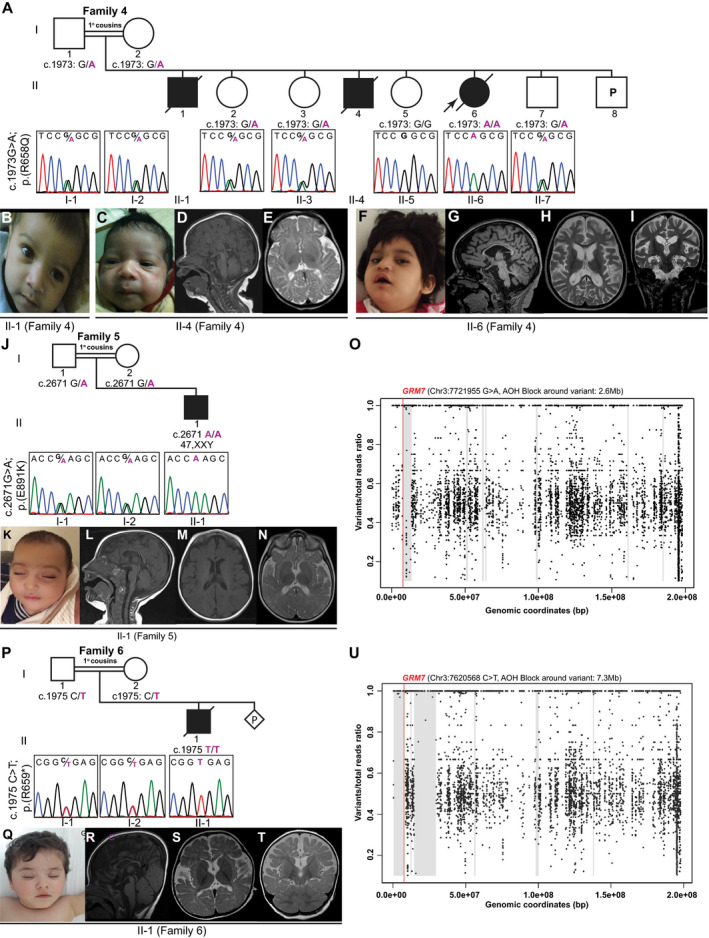
Pedigrees, Sanger sequencing, facial features, and brain MRI images of affected individuals from Families 4–6 with biallelic variants in *GRM7*. (A) Pedigree and Sanger sequencing showing segregation of the variants in *GRM7* in Family 4. (B) Facial photograph of individual II‐1 (Family 4) in first year of life with normal facial features. (C) Facial photograph of individual II‐4 (Family 4) at 1 month shows a long philtrum. (D and E) Brain MRI of individual II‐4 (Family 4) at 2 weeks. T1‐weighted image (midsagittal view) shows normal cerebrum, CC, and cerebellum (D). T2‐weighted image (axial view) shows under‐opercularization of sylvian fissures (more prominent on the left) (E). (F) Facial photograph of individual II‐6 (Family 4) at 4 years with hypotonic face and tented mouth. (G–I) Brain MRI of individual II‐6 (Family 4) at 4 years. T1‐weighted image (midsagittal view) shows severe cerebral atrophy, severe thinning of CC, and mild cerebellar atrophy (G). T2‐weighted images (axial and coronal views) show severe cerebral atrophy, severe thinning of CC, and bilateral hippocampal atrophy with right hippocampal hyperintensity (H and I). (J) Pedigree and Sanger sequencing showing segregation of the variants in *GRM7* in Family 5. (K) Facial photograph of individual II‐1 (Family 5) at 7 months showing normal facial features. Note that individual II‐1 (Family 5) carries a second molecular diagnosis of Klinefelter syndrome as his chromosomal analysis showed 47,XXY. (L‐N) Brain MRI of individual II‐1 (Family 5) at 6 months. T1‐weighted image (midsagittal view) shows thin CC (L). T1‐wieghted and T2‐weighted images (axial views) show simplified gyral pattern (M‐N). (O) B‐allele frequency for individual II‐1 (Family 5) calculated from exome variant data demonstrates a 2.6 Mb block of AOH on chromosome 3, marked by grey zones, and show that *GRM7* is located within the AOH block. (P) Pedigree and Sanger sequencing showing segregation of the variants in *GRM7* in Family 6. (Q) Facial photograph of individual II‐1 (Family 6) at 20 months of age shows upslanted palpebral fissures. (R‐T) Brain MRI of individual II‐1 (Family 6) at 18 months. T1‐weighted image (midsagittal view) shows severe thinning of CC, moderate cerebral atrophy and mild cerebellar atrophy (R). T2‐weighted images (axial and coronal views) show moderate cerebral atrophy, global hypomyelination for age, and under‐opercularization of sylvian fissures (S and T). (U) B‐allele frequency for individual II‐1 (Family 6) demonstrates that *GRM7* is located within a large AOH block (7.3Mb) on chromosome 3 marked by grey zones.

By performing family‐based exome sequencing and rare variant filtering on a NDD cohort of 220 families from consanguineous populations from Saudi Arabia and Turkey, and using our previously described variant parsing and prioritization workflow,^21^ we identified four individuals (BAB6708, BAB6709, BAB10502, and BAB10517) from three unrelated families (Families 1, 5, and 6 in Figs. 1 and 2) with rare homozygous *GRM7* variants*.* Comprehensive analysis of all exome sequencing data was performed to identify rare, potentially damaging variants. No candidate variants in known disease genes were identified in any of the four individuals to provide an alternative genetic etiology.

Gene query to the Baylor Genetics diagnostic laboratory revealed five additional individuals (BAB8506, brother of BAB8506, BAB13620, 2 brothers of BAB13620) from two unrelated families (Families 2 and 4 in Figs. 1 and 2) with a similar neurological phenotype and deleterious biallelic *GRM7* variants detected on proband exome sequencing. Rudimentary clinical data were previously published on four of these individuals (BAB6708, BAB6709, BAB8506, and brother of BAB8506) from two families (Family 1 and Family 2) as part of a cohort neurological study.^19^ Two additional subjects from one family (MR005, Family 3) were identified by literature search, also published with limited phenotypic data in a gene discovery cohort.^20^ Gene submission to GeneMatcher re‐captured Family 3 (MR005).^22^

In Family 3 (MR005), linkage analysis and homozygosity mapping were performed using HumanCytoSNP‐12 BeadChip of all available members of the family (proband, both parents, and five unaffected siblings) as described in Abou Jamra *et al.* (2011).^23^ In total, five regions of 1Mb or more (between 2.6 and 9.8 Mb, total length of 24.6 Mb) with runs of homozygosity (ROH) resulting in autozygosity were identified. Evaluation of the variants was performed based on zygosity and locus (in linkage regions) as previously described,^24^ coupled with variant prevalence in public and private databases and prediction models as described in the cases below.

All seven identified *GRM7* variants were absent in the homozygous state from public variant databases including the Genome Aggregation Database (gnomAD), the Exome Aggregation Consortium (ExAC), the Atherosclerosis Risk in Communities Study Database (ARIC), and the National Heart, Lung, and Blood Institute (NHLBI) Grand Opportunity Exome Sequencing Project (ESP) as well as from our in‐house control database. The variants were also absent in heterozygous and homozygous state from the Iranome database (http://www.iranome.com) that contains exomes of 100 healthy individuals from related ethnic groups.^25^ Three variants (RefSeq: NM_000844.4; c.461T>C, c.1972C>T, and c.2024C>A) were entered by our group into a public archive of human variation and phenotypes (ClinVar) following the publication of the initial cohort (ClinVar accession numbers VCV000242895, VCV000242900, and VCV000242901, respectively).^21^ Bioinformatic analyses (SIFT, PolyPhen2, MutationTaster, CADD, and PhyloP) were used to predict the potential deleterious or pathogenic effect of variants on protein function and also evolutionary conservation. In four subjects (Family 1 individuals II‐1 and II‐2, Family 5 individual II‐1, and Family 6 individual II‐1), absence of heterozygosity (AOH) was determined based on the calculated B‐allele frequency from exome data using an in‐house developed bioinformatic tool, BafCalculator (https://github.com/BCM-Lupskilab/BafCalculator), as previously described.^26^ An arbitrary cutoff point or value of 0.5 Mb was used in calculating the size of the AOH, and unphased data or ROH block around the variant as well as the total genomic AOH/ROH. In one subject (Family 4 individual II‐6), ROH was calculated from SNP array data.

Variants in all affected individuals, their parents, and any available unaffected siblings were verified by Sanger sequencing for segregation studies. Molecular diagnosis was confirmed in 8 of the 11 affected individuals that were alive at the time of the study, although all 11 subjects were clinically examined during the course of their illness for the features documented. Referring physicians provided detailed clinical information and assessments on all subjects for deep phenotyping. Updated clinical history and clinical pictures were obtained on two of three previously published families (Families 1 and 2).^19^ Brain magnetic resonance images (MRIs) of eight individuals were reviewed by a board‐certified neuroradiologist (JVH). Electroencephalograms (EEGs) of two individuals were reviewed by a board‐certified clinical neurophysiologist (DM).

### 3D modeling of protein structure

3D protein structure of GRM7 was obtained from SWISS‐MODEL homology modeling server.^27^ This crystal structure covers amino acids from 41 to 849. Protein structure was displayed by the PyMol Molecular Graphics System, v.1.5. Schrodinger, LLC. Amino acid conservation was obtained from the Consurf server based on sequence analysis.^28^

## Results

### Clinical findings

We performed a comprehensive retrospective analysis of patients’ clinical data including birth and perinatal history, age of onset of disease, neurological features, seizure types and response to treatment, growth parameters, neurological exam findings as well as diagnostic studies including EEGs and brain MRIs.

Basic clinical information and perinatal history of the 11 affected individuals from six unrelated families with deleterious biallelic *GRM7* variants are summarized in Table 1. Subjects were from diverse ethnic backgrounds and countries of origin including Saudi Arabia, Syria, Turkey, United Arab Emirates, and the United States. All individuals were born at term. Prominent perinatal complications included neonatal intensive care unit (NICU) admission for respiratory distress, apnea, desaturation or neonatal seizures in five individuals (Family 1 individual II‐1, Family 2 individual II‐2, and Family 4 individuals II‐1, II‐4, and II‐6), and polyhydramnios in four individuals (Family 4 individuals II‐1, II‐4, and II‐6 and Family 5 individual II‐1). No history of perinatal insult was found in any individuals. Birth weights were available in nine individuals and were normal and within one standard deviation (SD) of the mean for age. Head circumference measurement at birth was only available in one subject (Family 2 individual II‐2) and was also normal while head circumference measurements at birth were not available for the remaining subjects.

**Table 1:** Basic clinical information and perinatal history of families with biallelic *GRM7* variants.

Individual	Country of origin	Sex	Current age	Gestational age at birth/mode of delivery	Perinatal complications	OFC‐birth/cm (*z*‐score)	Birth weight/gram (*z*‐score)
Family 1, II‐1 (BAB6709)*	Saudi Arabia	M	13 years	FT (39 w)/CS	Decreased fetal movements, oligohydramnios, and fetal bradycardia requiring emergency CS; NICU admission for respiratory distress (on mechanical ventilation)	N/A	3000 g (−0.9 SD)
Family 1, II‐2 (BAB6708)*	Saudi Arabia	M	10 years	FT (39 w)/CS	None	N/A	3000 g (−0.9 SD)
Family 2, II‐1 (BAB8506)*	USA	F	15 years	FT (38 w)/SVD	None; failed hearing screen one side	N/A	3800 g (+0.82SD)
Family 2, II‐2 (BAB8506's brother)*	USA	M	10 years	FT (39 w)/SVD	NICU admissions neonatal seizures at 12 HOL	36 cm (+0.06SD)	3700 g (+0.28 SD)
Family 3, II‐2 (MR005‐1)*	Syria	F	Died at 13 years (poor nutritional status)	FT/SVD	None	N/A	N/A
Family 3, II‐6 (MR005‐2)*	Syria	M	N/A	FT/SVD	None	N/A	N/A
Family 4, II‐1	UAE	M	Died at 13 months (respiratory failure)	FT/CS	Polyhydramnios, NICU admission for respiratory distress and desaturations	N/A	3500 g (−0.10 SD)
Family 4, II‐4	UAE	M	Died at 45 days (SIDS)	FT (41 w)/SVD	Polyhydramnios, NICU admission for respiratory distress and seizures	N/A	3700 g (+0.28 SD)
Family 4, II‐6 (BAB13620)	UAE	F	Died at 4 years (aspiration PNA)	FT (41 w)/CS	Polyhydramnios and GDM, maternal influenza, and NICU admission for apnea, desaturations, hypoglycemia, and jaundice	N/A	3200 g (−0.43 SD)
Family 5, II‐1 (BAB10502)	Turkey	M	2 years 2 months	FT/SVD	Polyhydramnios and GDM	N/A	3700 g (+0.28 SD)
Family 6, II‐1 (BAB10517)	Turkey	M	Died at 5 years (aspiration PNA)	FT/SVD	None	N/A	3300 g (−0.42 SD)

CS, C‐section; F, female; FT, full term; g, grams; GDM, gestational diabetes mellitus; HOL, hours of life; M, male; N/A, not available; NICU, neonatal intensive care unit; OFC, occipital frontal circumference; PNA, pneumonia; SIDS, sudden infant death syndrome; SD, standard deviation; SVD, spontaneous vaginal delivery; w, weeks; UAE, United Arab Emirates; USA, United States of America.

* Previously published with limited clinical data.

**Figure 3:**
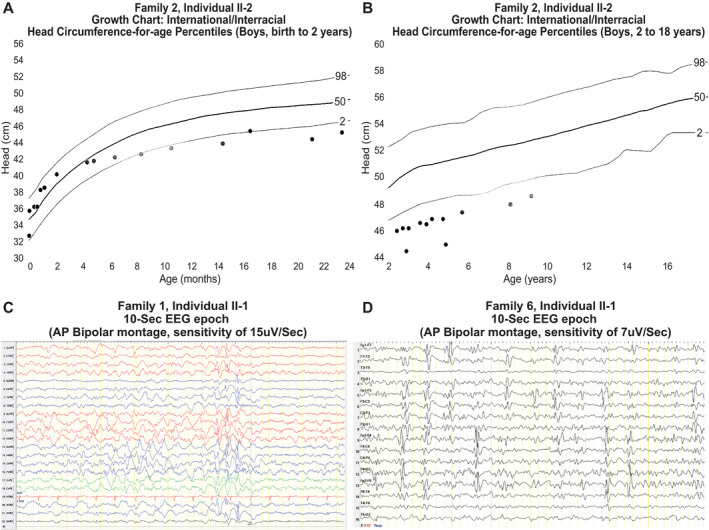
Head circumference charts and EEG epochs. (A) Head circumference‐for‐age chart (boys, birth to 2 years) of individual II‐2 from Family 2 showing normal head circumference at closer to 50th centile with crossing of centiles to less than 2nd centile starting at around 4 months of age through 14 months of age. The pattern is consistent with an acquired microcephaly. (B) Head circumference‐for‐age chart (boys, 2–18 years) of same individual (individual II‐2 from Family 2) showing that despite steady head growth, head circumference remains consistently under the 2nd centile from 2 years to 9 years of age. (C) 10‐sec epoch from a routine EEG of individual II‐1 from Family 1 (anterior–posterior bipolar montage, sensitivity of 15 *μ*V/Sec) showing diffuse background slowing and multifocal epileptiform activity most prominent at 6–7 sec from start of epoch. (D) 10‐se epoch from a routine EEG of Individual II‐1 Family 6 (anterior–posterior bipolar montage, sensitivity of 7 *μ*V/Sec) showing diffuse background slowing and abundant multifocal epileptiform activity throughout the epoch.

Neurological and other clinical features are detailed in Table 2. Shared clinical features in all 11 subjects include severe to profound global developmental delays (GDD), intellectual disability (ID), and early‐onset seizures within the first year of life. Head circumference measurements at time of last visit were available in eight individuals and were consistent with microcephaly (−3.8 to −2.7 SD from mean for age). Review of the head circumference chart from birth to current age in one individual (Family 2 individual II‐2) revealed an acquired microcephaly pattern with stagnation of head growth and crossing of centiles between 4 and 14 months (Fig. 3A and B). Other common neurological features included axial hypotonia (8/8), peripheral hypertonia (7/8), hyper‐reflexia (4/6), and drug‐resistant epilepsy (6/9). Status epilepticus was reported in four affected individuals (4/8). Additional clinical features include failure to thrive (6/11), recurrent infections (6/11), short stature (5/11), dysphagia (4/11), and cortical visual impairment (3/11). Hormonal problems were present in 4/11 individuals in the form of panhypopituitarism (1/11), growth hormone deficiency (1/11), hypothyroidism (1/11), or unspecified hormonal deficiency (1/11). Review of brain MRI images confirmed the absence of a structural pituitary abnormality in 3/4 of these subjects. The brain MRI images were not available for review in the subject with the unspecific hormonal deficiency. Occasionally reported medical problems include iron or B12 deficiency anemia (3/11), lipodystrophy (2/11), and sensory or conductive hearing loss (2/11). One subject had a nasogastric tube for feeding during infancy due to poor feeding and evidence of aspiration on modified barium swallow assessment. Self‐mutilation was reported in one subject. Four subjects died in infancy or early childhood due to either aspiration pneumonia (2/4), respiratory failure (1/4), or sudden infant death syndrome (1/4), and one individual died in late childhood (at 13 years) from poor nutritional status and lack of access to advanced medical care (Table 1).

**Table 2:** Neurological, developmental, and other clinical features of families with biallelic *GRM7* variants.

Individual	Age at last exam	OFC‐last exam cm (z‐score)	Microcephaly	Axial hypo‐tonia	Peripheral hyper‐tonia	Hyper‐reflexia	DD/ID	Seizures (onset)	DRE (Current AEDs)	Seizure types	SE	Other clinical features
Family 1, II‐1 (BAB6709)*	13 yrs.	50 cm (−2.9SD)	+	+	+	+	+	+ (4 mo.)	− (LEV and RFM‐effective)	Myoclonic	−	FTT, RI, dysphagia, anemia (B12 & iron deficiency), hypothyroidism, bilateral hyperopia, and occasional self‐mutilation
Family 1, II‐2 (BAB6708)*	10 yrs.	N/A	N/A	+	+	+	+	+ (8 mo.)	− (none)	GTCs (seizure free off AEDs for 5 yrs with subsequent recurrence)	−	N/A
Family 2, II‐1 (BAB8506)*	15 yrs.	50.5 cm (−3.31SD)	+	+	+	−	+	+ (1 mo.)	+ (LEV and VNS)	Myoclonic & GTCs	+ (one admission)	FTT, SS, dysphagia, cortical visual impairment, GH deficiency, hypotonic facies, vitamin D deficiency, low bone density, and stool incontinence
Family 2, II‐2 (BAB8506's brother)*	10 yrs.	48.6 cm (−3.29SD)	+	+	+	N/A	+	+ (DOL1)	+ (LEV, ZNS, and VNS)	Myoclonic & GTCs	−	FTT, SS, dysphagia, cortical visual impairment, hypotonic facies, and micropenis
Family 3, II‐2 (MR005‐1)*	N/A	N/A (<−2 SD)	+	N/A	N/A	N/A	+	+	N/A	N/A	N/A	FTT, RI, SS, and lipodystrophy
Family 3, II‐6 (MR005‐2)*	5 yrs. 6 mo.	N/A (<−2 SD)	+	N/A	+	N/A	+	+ (3 w)	N/A	N/A	N/A	FTT, RI, SS, “hormonal deficiency,” and lipodystrophy
Family 4, II‐1	13 mo.	N/A	N/A	+	N/A	N/A	+	+ (3 mo.)	+ (VPA and PB)	N/A	+ (ICU admission at 8 mo)	Dysphagia (nasogastric tube feeding), hepatomegaly, and an episode of hypoglycemia
Family 4, II‐4	1 mo.	N/A	N/A	N/A	N/A	N/A	+	+ (1 w)	−	N/A	N/A	N/A
Family 4, II‐6 (BAB13620)	3 yrs. 3 mo.	42.5 (−3.8 SD)	+	+	−	−	+	+ (5 mo.)	+ (VPA, LEV, and CLB)	GTCs & focal	−	RI, SS, cortical visual impairment, panhypopituitarism on hydrocortisone and thyroid hormone replacement, mild peripheral pulmonary stenosis, conductive hearing loss, CSA, ASD, concern for long QT syndrome, and HIE following aspiration at 3y 3m
Family 5, II‐1 (BAB10502)	7 mo.	40 cm (−3.2 SD)	+	+	+	+	+	+ (2 mo.)	+ (PB, CLB, LEV)	Myoclonic & GTCs	+ (2 admissions)	RI, OSA, anemia, and unilateral hearing loss
Family 6, II‐1 (BAB10517)	20 mo.	44.5 cm (−2.7 SD)	+	+	+	+	+	+ (2d)	+ (TPM, LMG, VPA, and CLB)	Multifocal	+ (multiple ICU admissions)	FTT, RI, and anemia
Total			8/8	8/8	7/8	4/6	11/11	11/11	6/9		4/8	

ADHD, attention deficit hyperactivity disorder; AED, antiepileptic drug; ASD, atrial septal defect; CLB, clobazam; CSA, central sleep apnea; DD, developmental delay; DOL, date of life; DRE, drug‐resistant epilepsy; FTT, failure to thrive; GH, growth hormone; GTC, generalized tonic‐clonic; HIE, hypoxic ischemic injury; ICU, intensive care unit; ID, intellectual disability; mo., months; LEV, levetiracetam; LMG, lamotrigine; N/A, not available; OSA, obstructive sleep apnea; PB, phenobarbital; RI, recurrent infections; SE, status epilepticus; SS, short stature; TPM, topiramate; VNS, vagal nerve stimulator; VPA, valproic acid, w, weeks; yrs., years; ZNS, zonisamide.

* Previously published with limited clinical data.

Neuroimaging studies of 10 affected individuals are summarized in Table 3, and representative images are displayed in Figures 1 and 2. Neuroimaging included brain MRIs in nine subjects and a head CT in one additional individual. Prominent neuroimaging findings included cerebral atrophy (8/10) that ranged from mild (2/8) to moderate/severe (6/8), global hypomyelination in 8/10 individuals, and moderate or severe thinning of corpus callosum (CC) in 7/8. Additional findings included mild cerebellar atrophy in 4/10 individuals, under‐opercularization of the Sylvian fissures in 3/10, and hippocampal atrophy or signal abnormality in 2/9, and brainstem volume loss in one individual (Family 4 individual II‐6).

**Table 3:** Radiological, electrographic, and other significant laboratory findings in families with biallelic *GRM7* variants.

Individual	Family 1, II‐1 (BAB6709)*	Family 1, II‐2 (BAB6708)*	Family 2, II‐1 (BAB8506)*	Family 2, II‐2 (BAB8506's brother)*	Family 3, II‐2 (MR005‐1)*	Family 3, II‐6 (MR005‐2)*	Family 4, II‐1		Family 4, II‐4	Family 4, II‐6 (BAB13620)	Family 5, II‐1 (BAB10502)	Family 6, II‐1 (BAB10517)	Total
Electroencephalogram (EEG) findings													
Diffuse background slowing	+	N/A	−	+ (also disorganized with lack of AP gradient)	N/A	N/A	+ (also attenuated)	−		+	+	+	6/8
Epileptiform activity	+ (frequent to abundant, multifocal over LPO, L hemispheric, LFC, LF, LT> R hemispheric)	N/A	+ (occasional, sleep‐activated, focal over LT and RF regions	+ (frequent, multifocal over RF, LF, and BiO regions)	N/A	N/A	+ (occasional, multifocal)	−		+ (frequent, high voltage 1‐2 Hz GSSW)	+ (focal, LFC with generalized spread)	+ (frequent, multifocal over RFC, RT, LF, and LP)	7/8
Ictal findings	+ (2 clinical generalized myoclonic seizures with generalized electro decrement; 6 electrographic seizures)	N/A	−	−	N/A	N/A	−	−		+ (7 clinical focal tonic seizures with generalized electro decrement; 1 electrographic seizure)	−	−	2/8
Type of neuroimaging (age)	Brain MRI (3 years)	Brain MRI (2 years)	Serial brain MRIs (2 mo, 18 mo, 3 yrs, 7 yrs & 10 yrs)	Serial brain MRIs (DOL2, 2 yrs & 5 yrs)	N/A	Brain MRI (N/A)	CT head (2 mo)	Brain MRI (2 w)		Brain MRI (3 yrs)	Brain MRI (6 mo)	Brain MRI (18 mo)	
Neuroimaging findings													
Cerebral atrophy	++/+++	++/+++	+	+	N/A	+++	+++	−		+++	− (simplified gyri)	++	8/10
Hypomyelination	+	−	++	++	N/A	+	+	−		+	+	+	8/10
CC thinning	++/+++	+++	+++	+++	N/A	N/A	N/A	−		++/+++	+++	+++	7/8
Cerebellar atrophy	+	−	−	−	N/A	+	−	−		+	−	+	4/10
Under‐opercularization of sylvian fissures	−	−	−	−	N/A	−	−	+		−	+	+	3/10
Hippocampal abnormality	−	+ (b/l T2 hyper‐intensity)	−	−	N/A	N/A		−		+ (b/l hippocampal atrophy with R hippocampal T2 hyperintensity)	−	−	2/9

AP, anterior–posterior; BiO, Bi‐occipital; b/l, bilateral; CC, corpus callosum; DOL, day of life; GSSW, generalized slow spike wave; indiv., individual; L, left; LF, left frontal; LFC, left frontocentral; LP, left parietal; LPO, left parieto‐occipital; LT, left temporal; mo, months; MRI, magnetic resonance imaging; N/A, not available; R, right; RF, right frontal; RT, right temporal; w, weeks; yrs, years. Note that (− and +) is used to indicate the absence or presence of the finding, respectively. Degree of presence is reflected by number of (+) signs, where one is used for mild, (++) is used for moderate and (+++) is used for severe.

* Previously published with limited clinical data.

One subject (Family 4 individual II‐6) had a complicated medical course and developed hypoxic ischemic encephalopathy (HIE) at age 3 years and 3 months following an aspiration event during oral feeding that led to cardiac asystole and respiratory failure with return of circulation after 30 min of cardiopulmonary resuscitation. A brain MRI obtained 1 week following the aspiration event showed interval worsening of the previously identified cerebral volume loss and atrophy.

Serial brain MRIs were available in two siblings (Family 2 Individuals II‐1 and II‐2) from birth or 2 weeks of age until the ages of 10 and 5 years, respectively. Of note, in both siblings, the myelination and CC were normal in the first 2 months of life but showed marked CC atrophy and loss of myelination at follow‐up brain MRI during the second year of life with plateauing of myelination and an apparent static myelin state on brain MRIs obtained following the second year of life (Fig. 1L–Q and S–X). One of these siblings (Family 2 Individual II‐2) had documented acquired microcephaly with onset of 4 months as discussed above (Fig. 3A and B). These findings suggest that the natural course of disease is progressive in the first 2 years of life followed by a static course.

Brain magnetic resonance spectroscopy (MRS) was performed in only one individual (Family 4 individual II‐6) and was obtained at 6 months of age revealing a markedly reduced N‐acetyl aspartate level and relatively high choline peak. No glutamate peak was noted. Additionally, cerebrospinal fluid (CSF), glucose, lactate, and neurotransmitter metabolites were performed in two affected individuals (Family 2 individual II‐2 and Family 4 individual II‐6) and were all within normal limits. One of these subjects (Family 2 individual II‐2) also had a normal CSF amino acids profile.

EEG findings are summarized in Table 3 with available EEG traces shown in Figure 3. The majority of the subjects (6/8) had diffuse background slowing indicating cerebral dysfunction. Epileptiform activity was also present in almost all subjects (7/8), with focal or multifocal epileptiform activity (6/8) seen more frequently than a generalized slow spike and slow wave pattern (1/8). Seizures captured on EEGs in two subjects included generalized myoclonic seizures with generalized electro‐decrement and superimposed paroxysmal fast activity in one subject, and focal tonic with generalized electro‐decrement in the second subject. The presence of severe developmental impairment, early‐onset seizures and frequent epileptiform activity with background slowing on EEG in a majority of subjects is consistent with DEE.

### Molecular characterization of rare *GRM7* biallelic variants

**Figure 4:**
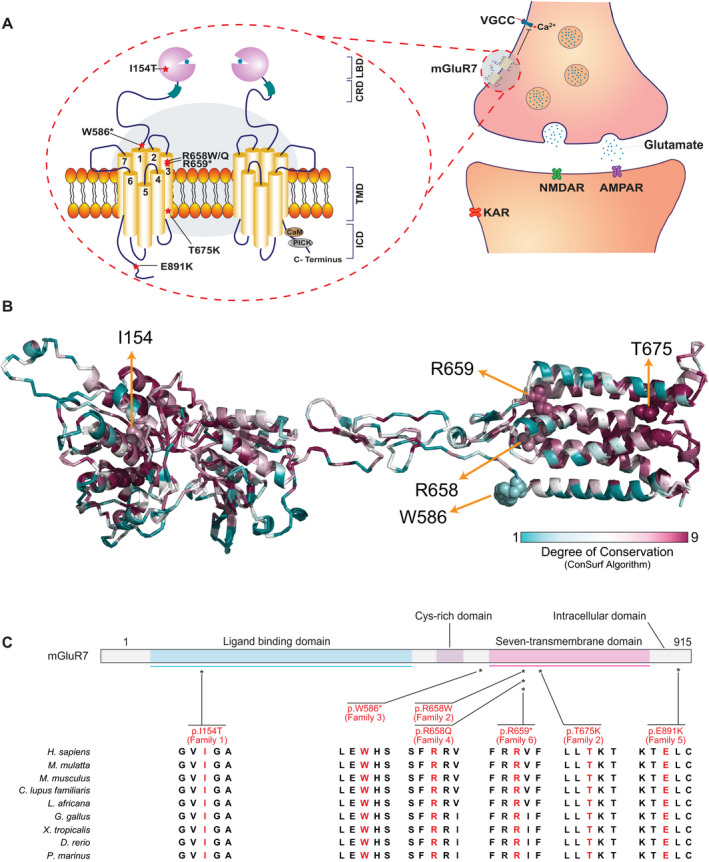
Variant locations on mGluR7 schematic and protein model and their conservation. (A) Schematic diagram of the neuronal synapse showing the presynaptic location of mGluR7 and the postsynaptic location of the iGluR7 including NMDAR, AMPAR, and KAR. A magnified illustration of mGluR7 shows that mGluR7s function as constitutional dimers and consist of four regions: N‐terminal ligand‐binding domain (LBD), cysteine‐rich domain (CRD), transmembrane domain (TMD), and intracellular domain (ICD). The approximate location of the altered amino acid residues described in this paper is displayed on the mGluR7 on the left. (B) Model protein structure of *GRM7*. The model structure covers amino acids from 41 to 849. The locations of the altered amino acids are represented by spheres on the protein cartoon model. I154 is buried on a loop between highly conserved alpha helices and beta sheets. Amino acid residues R658, R659, and T675 are highly conserved and located on the same helix of on transmembrane domain, while W586 is less conserved and located on the loop of the transmembrane domain. Note that E891 is not shown here, since the model structure does not include the intracellular region much. (C) Structure of the mGluR7 protein and the position of the altered amino acid residues (I154T, W586*, R658W/Q, R659*, T675K, and E891K) and the evolutionary conservation of the altered amino acids is also displayed.

Table 4 contains the summary of the variants observed: seven variants from six unrelated families that included five homozygous variants and one family with compound heterozygous variants. The pedigree diagrams, Sanger sequencing data and segregation studies of the variants, and facial morphological features of the affected individuals are shown in Figures 1 and 2. mGluR7 protein model structure, localization of variants and implicated amino acids, and variant conservation are displayed in Figure 4. The majority of the identified variants cluster within the transmembrane domain, except for two variants that are located at the ligand‐binding or intracellular domains (Fig. 4A and C). All variants were located within highly conserved regions in vertebrates and higher species (Fig. 4B and C). In general, subjects with the stop‐gain or missense variants within the transmembrane domain (Families 2–4 and 6) showed a more severe phenotype compared to those with missense variants located within the ligand‐binding (Family 1) or intracellular (Family 5) domains. Compound heterozygous variants were present in one family (Family 2) with no reported history of consanguinity, while homozygous variants were present in the other five families (Families 1 and 3–6) with self‐reported history of consanguinity (1^o^ cousin marriages). All *GRM7* homozygous variants were located within large blocks of AOH ranging from 2.6 to 9.7Mb. Total AOH in these affected individuals ranged from 24.6 to 575Mb (Table 4). One subject (Family 5 individual II‐1) carried a second molecular diagnosis consistent with Klinefelter syndrome based on a clinical G‐banded chromosomal analysis showing 47,XXY. The diagnosis of Klinefelter syndrome in this subject may contribute to the NDD phenotype but does not fully explain the severe neurological features in isolation.

**Table 4:** Summary of *GRM7* variants.

Individual	Position (GRCh37\hg19)	Nucleotide (Protein)	Zygosity	Allele count/Zygosity (gnomAD)	CADD Score (PHRED)	Conservation (phyloP)	Conservation (ConSurf algorithm)	AOH region around gene (Mb)	Total AOH (Mb)
Family 1, II‐1 (BAB6709)*	Chr3:6903536; T>C	c.461T>C (p.I154T)	Hmz	0 Htz–0 Hmz	28.9	1.978	7	8.7	575
Family 1, II‐2 (BAB6708)*	Chr3:6903536; T>C	c.461T>C (p.I154T)	Hmz	0 Htz–0 Hmz	28.9	1.978	7	9.7	411
Family 2, II‐1 (BAB8506)*	Chr3:7620565; C>T	c.1972C>T (p.R658W)	Comp Htz	0 Htz–0 Hmz	26.2	0.962	7	–	–
	Chr3:7620617; C>A	c.2024C>A (p.T675K)	Comp Htz	0 Htz–0 Hmz	26.1	6.104	9	–	–
Family 2, II‐2 (BAB8506's brother)*	Chr3:7620565; C>T	c.1972C>T (p.R658W)	Comp Htz	0 Htz–0 Hmz	26.2	0.962	7	–	–
	Chr3:7620617; C>A	c.2024C>A (p.T675K)	Comp Htz	0 Htz–0 Hmz	26.1	6.104	9	–	–
Family 3, II‐2 (MR005‐1)*	–	–	–	–	–	–			
Family 3, II‐6 (MR005‐2)*	Chr3:7620350; G>A	c.1757G>A (p.W586*)	Hmz	0 Htz–0 Hmz	51	N/A	3	3.7	24.6
Family 4, II‐1	–	–	–	–	–	–	–	–	–
Family 4, II‐4	–	–	–	–	–	–	–	–	–
Family 4, II‐6 (BAB13620)	Chr3:7620566; G>A	c.1973G>A (p.R658Q)	Hmz	5 Htz–0 Hmz	24.9	0.962	7	6	113
Family 5, II‐1 (BAB10502)	Chr3:7721955; G>A	c.2671G>A (p.E891K)	Hmz	5 Htz–0 Hmz	33	0.902	8	2.6	545
Family 6, II‐1 (BAB10517)	Chr3:7620568; C>T	c.1975C>T (p.R659*)	Hmz	1 Htz–0 Hmz	37	ND	8	7.3	361.2

AOH, absence of heterozygosity; CADD, Combined Annotation Dependent Depletion; Comp.Htz, compound heterozygous; Hmz, homozygous; Htz, heterozygous; N/A, not available; ND, not determined.

* Previously published with limited clinical data.

## Discussion

We comprehensively characterized the clinical features and variants in six families with biallelic variants in *GRM7* and showed that rare *GRM7* biallelic variants can cause a severe neurological phenotype characterized by microcephaly, DEE, hypomyelination, and cerebral atrophy. Overall, the findings of both congenital neuroimaging features such as under‐opercularization and thin corpus callosum and progressive features such as cerebral atrophy suggest that *GRM7*‐related disorders are both congenital and progressive in nature similar to that seen in *GRIA2‐*related NDDs.^7^ Additionally, other disorders such as *CDKL5*‐related and *FOXG1*‐related disorders cause a wide and heterogeneous range of NDDs.^29^, ^30^ It is possible that the phenotype‐first approach of our cohort has introduced an ascertainment bias toward enrolling subjects with more severe, and potentially homogenous, phenotype. Identification of more cases using a gene‐first approach will likely reveal the entire and broad spectrum of *GRM7*‐related disorders.^31^, ^32^

Genotype–phenotype correlation of our limited data suggests that stop‐gain and missense variants affecting the transmembrane domain result in a more severe phenotype compared to missense variants located in ligand‐binding or intracellular domains. We hypothesize that the reported biallelic variants are loss‐of‐function (LoF) alleles, with the missense variants causing partial LoF (hypomorphic) while the stop‐gain variants result in complete LoF (amorphic). Both stop‐gain variants identified (Family 3 and Family 6) were in exon 8 of total 10 exons in *GRM7*. The resultant transcripts with these premature termination codons will either be degraded by nonsense‐mediated decay or result in a truncated protein, both of which are expected to result in complete LoF.^33^ This LoF of mGluR7 is expected to result in loss of inhibition in the glutamatergic pathway, excess glutamate release, and subsequently hyperexcitability.

Glutamate is an excitatory neurotransmitter that is robustly implicated in the pathophysiology of epilepsy, a disorder characterized by aberrant neuronal hyperexcitability. Excessive and abnormal neuronal firing in seizures leads to depolarization shift, increase in extracellular glutamate, and subsequent neuronal excitotoxicity.^3^ This results in a vicious cycle with aberrant glutamate release contributing to further epileptogenicity, and seizure maintenance and progression.^3^

Hyperexcitability of the glutamatergic pathway could lead to the severe epilepsy phenotype as noted in all affected individuals. All our subjects had the onset of their seizures in infancy, with seizures starting in the neonatal period in nearly half of them (Table 2). Additionally, the significant history of early postnatal apneas and desaturations requiring NICU admission may suggest the possibility of missed seizures with earlier onset than reported in some individuals. Two of our subjects showed early signs of early hippocampal sclerosis (HS) with hippocampal atrophy and signal abnormality. While it remains unclear if HS is a cause or a sequela of frequent seizures, the aberrant expression of mGluR7 in the hippocampus may be a contributing factor.^34^, ^35^

Excessive glutamate is also neurotoxic, potentially explaining the marked and early progressive cerebral atrophy and axonal loss observed in our subjects, particularly the affected siblings in Family 2 with the observations of longitudinal MRI data and the acquired microcephaly pattern in one of them. Interestingly, postnatal deceleration of head growth has been observed in 5/28 subjects with deleterious monoallelic variants in *GRIA2*, a gene encoding a subunit of another glutamate receptor, AMPAR.^7^ However, our ability to further assess or quantify this finding is limited due to the lack of longitudinal data in other subjects. One caveat is that one of our subjects (Family 4 individual II‐6) had an MRS that did not show a glutamate peak, but this observation cannot be generalized either, as it was a one‐time observation in a single subject without longitudinal data.

At the neuronal synapses, glutamate signals through two groups of receptors, iGluRs and mGluRs. AMPAR is the most abundant iGluR in the mammalian brain and is responsible for fast excitatory neurotransmission while NMDAR is important for slow synaptic potential and information processing.^3^ KAR is thought to play a role in presynaptic and postsynaptic modulation of neurotransmission.^36^ *De novo* and inherited pathogenic variations in genes encoding postsynaptic iGluRs subunits cause a wide range of NDDs and DEEs.^4^, ^5^, ^6^, ^7^, ^8^, ^9^, ^10^ Interestingly, biochemical and in vitro functional assays show that variants in the same gene encoding some NMDAR and AMPAR subunits (e.g. *GRIN2A, GRIN2B, GRIN2D,* and *GRIA2*) may act as both gain‐of‐function (GoF) and LoF and may result in indistinguishable neurological phenotypes.^5^, ^6^, ^7^ This alludes to the complexity of the glutaminergic pathway with both enhanced and reduced function leading to NDDs and DEEs. Patho‐mechanisms include alterations in any of the multiple functional parameters including agonist potency, sensitivity to negative allosteric modulators, channel opening probability, surface expression, and current amplitude response.^6^, ^7^

mGluR7, encoded by *GRM7*, is the most abundant CNS mGluR and is only activated by high glutamate and GABA concentrations due to its low affinity for these neurotransmitters. This allows it to function as an auto or hetero‐receptor to downregulate further calcium‐dependent glutamate release and thus prevent the neurotoxic effect of extracellular glutamate accumulation.^37^ mGluR7 is evolutionarily conserved and is widely distributed across the CNS including the hippocampus, hypothalamus, and thalamo‐cortical circuitry synapses.^35^

Activation of mGluR7 produces a cascade of events starting with the liberation of Gβγ subunits to inhibition of adenylyl cyclase and reduced cAMP production and ending with the downregulation of voltage‐gated calcium channels (VGCC).^38^ mGluR7 also interacts with several intracellular and scaffolding proteins including PKC (protein kinase C) and PDZ domain‐containing protein PICK1 (protein interacting with C kinase 1), providing further complexity to the mechanisms by which it regulates synaptic transmission.^39^ The mGluR7‐PICK interaction is critical for receptor function, thus raising the possibility that the intracellular variant (p.Glu891Lys) in one of our subjects causes disease by disrupting this PICK‐mGlu7 interaction.

Rodent models of mGluR7 and its scaffolding (PICK1) or interacting proteins (ELFN1) show that mGluR7 reduced expression or impairment of its function or recruitment to synaptic sites lead to symptoms overlapping with NDDs and DEEs. mGluR7‐knockout mice exhibit spontaneous sensory‐provoked seizures; increased seizure vulnerability to two proconvulsant agents; reduced fear learning; signs of impaired learning, short‐term and spatial memory, and synaptic plasticity; and dysregulation of the hypothalamic–pituitary–adrenal (HPA) axis.^18^, ^40^, ^41^ Of note, HPA axis dysregulation was present in at least three of our subjects. Additionally, mice lacking transmembrane protein ELFN1 (extracellular‐leucine‐rich repeat fibronectin domain 1) show early postnatal deficits in recruiting mGluR7 to synaptic site and display a remarkably similar neurological phenotype to mGluR7‐deficient mice with late‐onset sensory‐triggered epileptic seizures and motor and behavioral abnormalities.^42^ Furthermore, impaired mGluR7 interaction with PICK1 by knockin PDZ‐recognition binding motif of mGluR7 or by pharmacological uncoupling in rodent models results in low seizure threshold to proconvulsive agents and produces behavioral changes and EEG discharges consistent with absence‐like seizures.^43^, ^44^ These animal models demonstrate that abnormal mGluR7 expression or function consistently produce an epilepsy phenotype.

Apart from one subject with a reported favorable response to rufinamide, we could not assess the response to each antiepileptic drug (AED) in retrospect, and our subjects had poor responses to AEDs overall (Table 2). However, several US Food and Drug administration (FDA)‐approved AEDs that reduce the glutaminergic pathway hyperexcitability already exist and show potential for use in such conditions. These AEDs exert their effect either directly or indirectly and may possess additional neuroprotective effect against glutamate excitotoxicity. Topiramate selectively inhibits the excitatory neurotransmission mediated by KAR and partially depresses AMPAR‐mediated excitatory current.^45^ Topiramate also shows a neuroprotective effect against glutamate excitotoxicity in rodent hippocampal neurons.^46^ Felbamate blocks NMDAR particularly at excessive exposure, thus inhibiting seizure discharges while preserving normal neuronal firing.^47^ Perampanel is a first‐in‐class noncompetitive selective AMPAR antagonist that blocks AMPAR by acting on an allosteric site rather than the glutamate recognition site.^48^ Voltage‐gated sodium channel blockers such as lamotrigine, phenytoin, carbamazepine, oxcarbazepine, lacosamide, rufinamide, and eslicarbazepine can also reduce glutamate release indirectly by blocking sodium‐evoked calcium influx.^49^ Gabapentin and pregabalin reduce the calcium‐dependent glutamate release by selectively binding at the α2δ‐1 subunit of VGCC.^50^ In addition, cannabinoids, a newly emerging therapy in treatment of refractory epilepsy, can indirectly modulate glutaminergic neurotransmission through neuronal cannabinoid receptor 1 (CB1).^51^ Whether any of the above‐mentioned AEDs that target the glutaminergic pathway prove to be particularly effective in treating epilepsy due to deleterious variants in *GRM7* is an area for further investigation.

Additionally, there are ample neuropharmacological studies focusing on the potential for using allosteric modulators of mGluRs in the treatment of neurological diseases and epilepsy.^52^, ^53^ Selective positive allosteric modulators (PAMs) for group III mGluRs have been recently developed, potentially representing novel targeted and personalized therapy in partial mGluR7 LoF (hypomorph alleles). PAMs are noncompetitive agonists that bind to a site other than the ligand‐binding site to potentiate their effect.^12^ N,N′‐dibenzhydrylethane‐1,2‐diamine dihydrochloride (AMN082) is the first selective mGluR7 PAM to be discovered.^54^ AMN082 binds to an allosteric site at the transmembrane domain to fully activate mGluR7.^54^ Thus, the potential use of AMN082 would be predicted to be dependent on the variant location. However, AMN082 shows scarce selectivity in vivo, off‐target effect, and mixed proconvulsive and anticonvulsive profile in pentylenetetrazol‐treated rats, limiting its potential for clinical use and underscoring a need for preclinical in vivo knockout studies.^55^, ^56^

We show here that rare biallelic variants in *GRM7* cause a severe neurological phenotype characterized by microcephaly, DEE, hypomyelination, and cerebral atrophy. Functional studies at the variant level would provide better insight into the patho‐mechanism of the disorder and evaluate the potential for targeted therapy.

## Conflict of Interest

J.R.L. has stock ownership in 23andMe, is a paid consultant for Regeneron Pharmaceuticals and Novartis, and is a co‐inventor on multiple United States and European patents related to molecular diagnostics for inherited neuropathies, eye diseases, and bacterial genomic fingerprinting. The Department of Molecular and Human Genetics at Baylor College of Medicine receives revenue from clinical genetic testing conducted at Baylor Genetics (BG) Laboratories; JRL is a member of the Scientific Advisory Board of BG Laboratories. Other authors have no potential conflicts to report. J.V.H receives royalties from chapter in UpToDate on pediatric neuroimaging.

## Authors’ Contribution

D.M., T.M., D.P., J.F, J.E.P., and J.R.L. contributed to the conception and design of the study. All authors contributed to the acquisition and analysis of data. D.M., T.M, D.P., J.F., H.D., S.E., J.V.H, J.E.P., and J.R.L drafted a significant portion of the manuscript or contributed to the design of the figures.
